# Supplementary material for: Insights into the structure-function relationship of the NorQ/NorD chaperones from Paracoccus denitrificans reveal shared principles of interacting MoxR AAA+/VWA domain proteins
Source: BMC Biol. 2023 Feb 28;21:47. doi: 10.1186/s12915-023-01546-w (PMC9976466; doi:10.1186/s12915-023-01546-w)
Supplement: Supplementary file 1 — Additional file 1: Fig S1. SEC profiles. Fig S2. Comparison of VWA domains and the MIDAS region from different organisms. Fig S3. 2D class averages of NorQ in cryo-EM experiments. Fig S4. Sequence alignment, characteristics and homology model of NorQ. Fig S5. Prediction of disordered regions and overall architecture for full-length NorD from P. denitrificans. Fig S6. Finger-like protrusions predicted for several MoxR-associated VWA domains and pairwise alignments. Fig S7. AlphaFold-multimer model of the full-length NorD in complex with the NorQ hexamer. Fig S8. Superposition of the NorQ-VWA AlphaFold prediction and the fitted cryo-EM-model. Fig S9. Extra densities in the NorQWBD cryo-EM map. Fig S10. ATPase activity measurements. Fig S11. Sequence alignment of the NorB subunit of cNOR with NorZ (qNOR) from various organisms. Fig S12. Predicted VWA domain of NorD in comparison to several eukaryotic VWA domains. Fig S13. Structural connection between the “finger” and the MIDAS site in the NorD VWA domain. Table S1. Mass spectrometry analysis. Table S2. AlphaFold-multimer prediction datasets. Table S3. Re-activation experiments with cNOR using crude cell extracts. Table S4. Primers used in this study. [file 12915_2023_1546_MOESM1_ESM.pdf]

Supporting information for:

Insights into the structure-function relationship of the NorQ/NorD chaperones from *Paracoccus denitrificans* reveal shared principles of interacting MoxR AAA+/VWA domain proteins

Maximilian Kahle[1,3, %*], Sofia Appelgren[1%], Arne Elofsson[1], Marta Carroni[1,2*] and Pia Ädelroth[1*]

[1]Department of Biochemistry and Biophysics, Stockholm University, SE-106 91 Stockholm, Sweden

[2]Swedish Cryo-EM Facility, Science for Life Laboratory Stockholm University, Solna, Sweden

[3]Current address: Department of Biochemistry, University of Potsdam, 14476 Potsdam, Germany

Contains: Figures S1-S13, Tables S1-S4

[%]Authors contributed equally

[*]Address correspondence to: Pia Ädelroth: pia.adelroth@dbb.su.se; Tel. +46-8-164183. Maximilian Kahle: maximilian.kahle@uni-potsdam.de. Marta Carroni: marta.carroni@dbb.su.se.

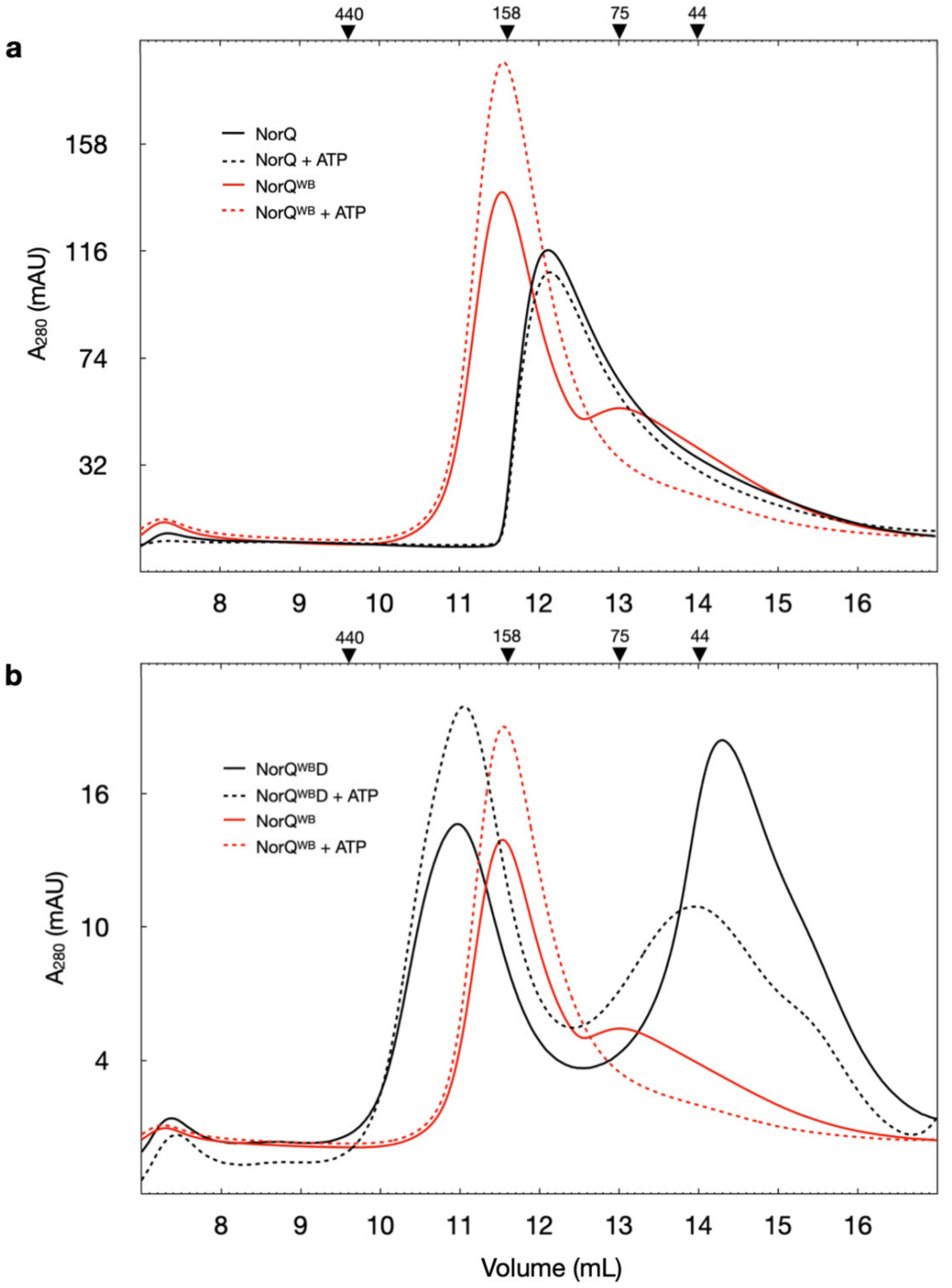

Figure S1: SEC profiles. *a) NorQ (black solid) and NorQ with 2 mM ATP (black dashed). Both show one major peak, eluting at ~120 kDa. NorQ^{WB} (red solid) showing two major peaks, eluting at ~160 kDa and ~70 kDa. NorQ^{WB} with 2 mM ATP (red dashed) showing only the ~160 kDa peak. b) NorQ^{WB}D (black solid) showing two major peaks, eluting at ~210 kDa and ~40 kDa and NorQ^{WB}D with 2 mM ATP (black dashed), also two major peaks at ~210 kDa and ~50 kDa. The same NorQ^{WB} profiles as in a) are shown for comparison. All samples were run on Superdex 200 10/300 GL and the markings on top are from a calibration with GE Gel Filtration HMW Calibration Kit.*

Table S1: Mass spectrometry analysis. *Selected bands from SDS-PAGE (see Figure 3c) were analyzed.*

**sample**	**detected protein**	**Nr. of identified peptides**	**coverage (%)**	**protein score**
**band 1**	*Major components:*			
	NorD	37	54	275
	NorQ	20	60	81
	E. coli contaminants:			
	Histone family protein DNA-binding protein	2	28	2
**band 2**	*Major components:*			
	NorQ	21	70	290
	NorD	32	53	84

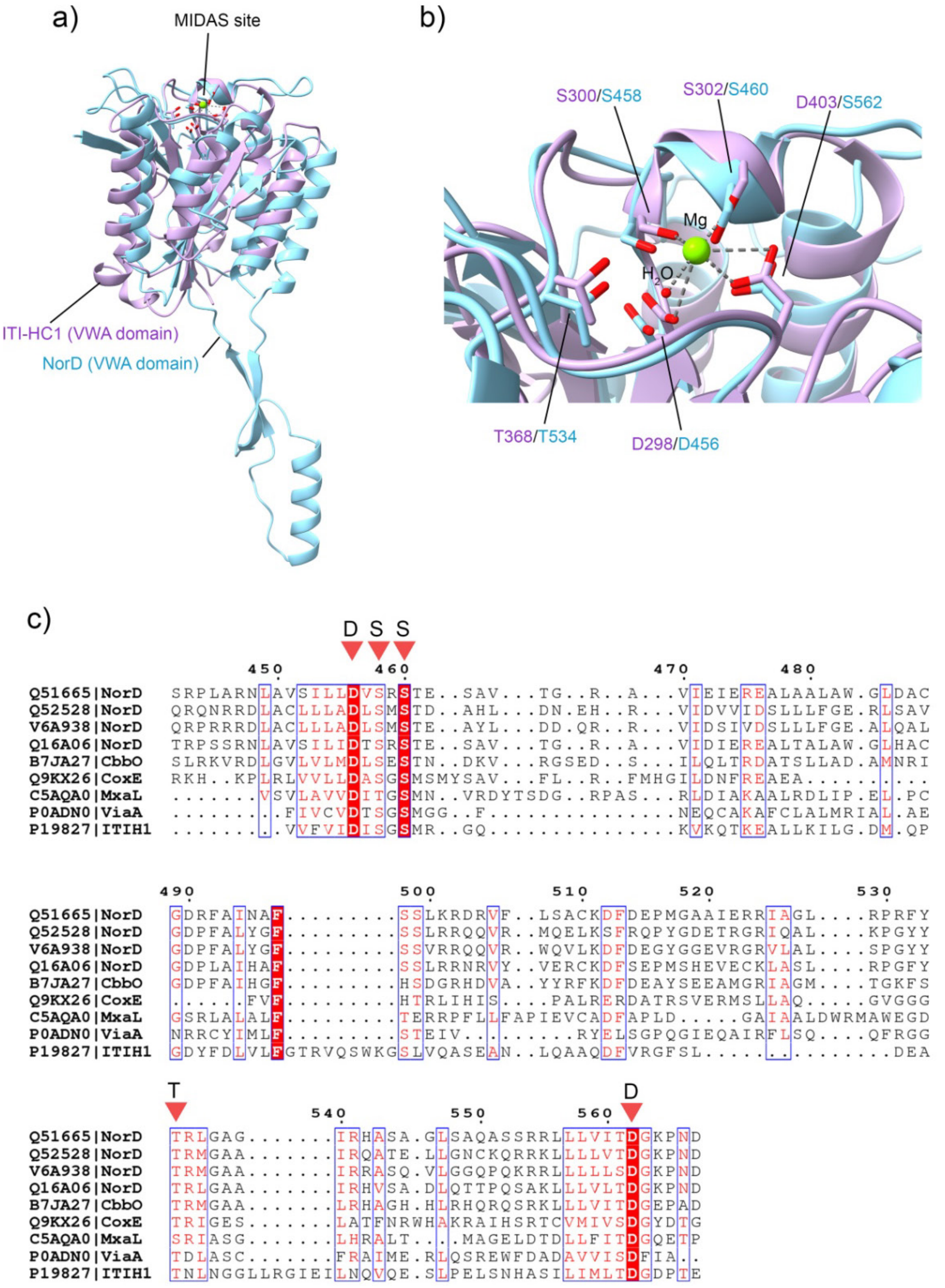

Figure S2: Comparison of VWA domains and the MIDAS region from different organisms. *a) Predicted AlphaFold VWA domain model of NorD (cyan, Uniprot (UP): Q51665, see also Fig. 7a in main text) superimposed on the crystal structure of the Inter-alpha-inhibitor heavy chain 1 (purple, ITI HC1, PDB ID: 6FPY). b) Enlargement of the conserved MIDAS region, with the residues shown to (for ITI, purple) or predicted to (for NorD, cyan) bind Mg (green) marked. c) Sequence alignment of selected VWA domain MIDAS regions: NorD from P. denitrificans (UP: Q51665), Ps. stutzeri (UP: Q52528), Ps. aeruginosa (UP: V6A938), and Roseobacter denitrificans (UP: Q16A06), CbbO1 from Acidithiobacillus ferrooxidans (UP: B7JA27), CoxE from Afipia carboxidovorans (UP: Q9KX26), MxaL from Methylorubrum extorquens (UP: C5AQA0), ViaA from E. coli (UP: P0ADN0) and human ITI-HC1 (UP: P19827). Residue numbers according to P. denitrificans NorD.*

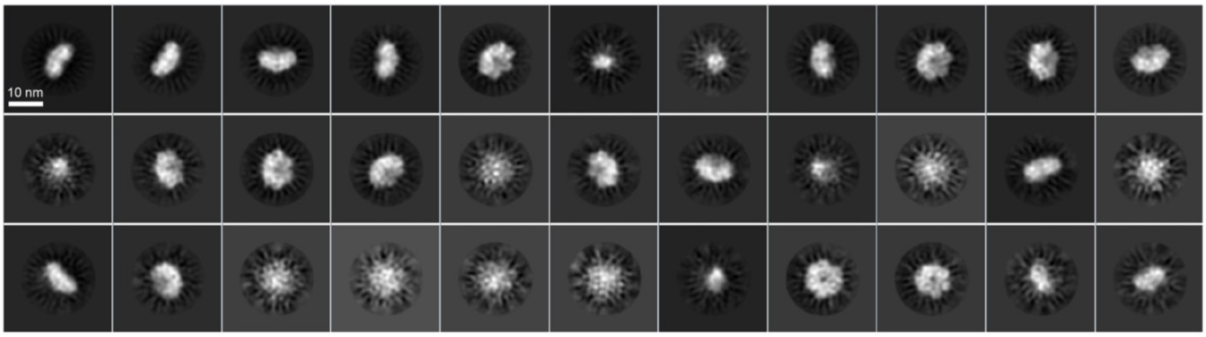

Figure S3: 2D class averages of NorQ in cryo-EM experiments. *The 2D classes represent 50,000 particles out of a total number of 130,000 particles.*

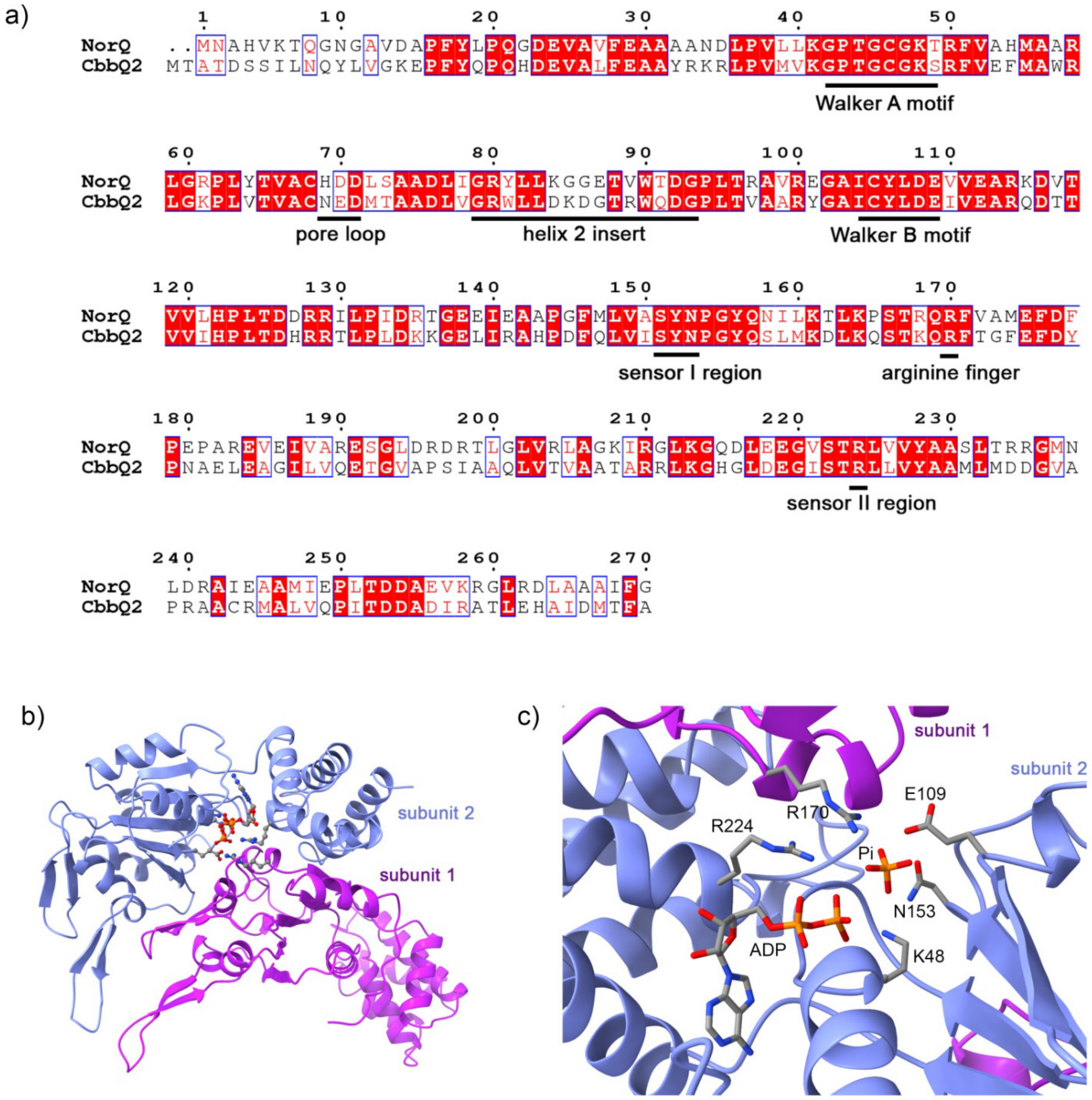

Figure S4: Sequence alignment, characteristics and homology model of NorQ. *a) Sequence alignment of AfCbbQ2 from A. ferrooxidans (Uniprot: B7J5E4) with NorQ from P. denitrificans (Uniprot: Q51664). The conserved features of the AAA+ domain are marked. b) Interaction of two adjacent NorQ subunits and formation of the active site (nucleotide and involved residues are shown in ball-stick representation). A homology model of the P. denitrificans NorQ is shown based on the crystal structure of AfCbbQ from A. ferrooxidans (PDB ID: 6L1Q). The coordinates of the nucleotide and phosphate are taken from the AfCbbQ crystal structure after superimposition with the NorQ model. c) Close-up of the nucleotide binding pocket of the NorQ model formed between neighboring subunits.*

a)

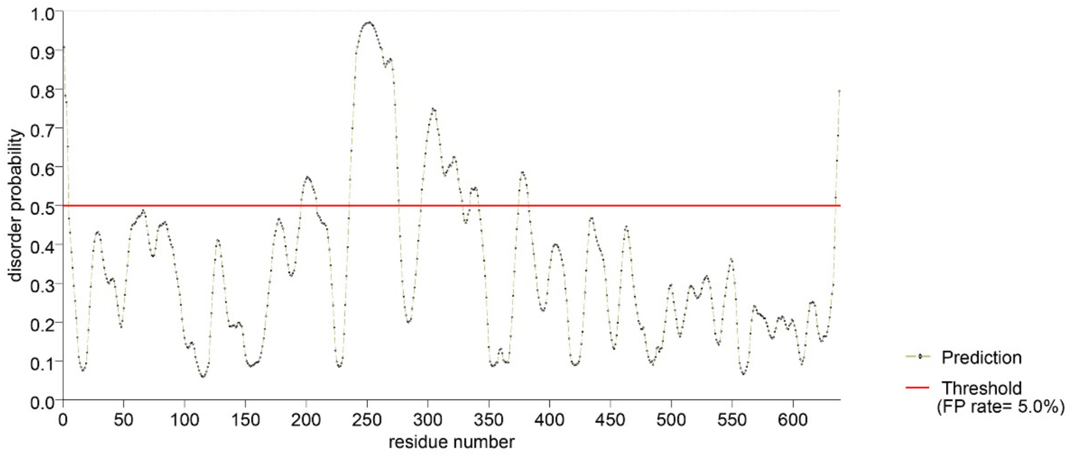

b)

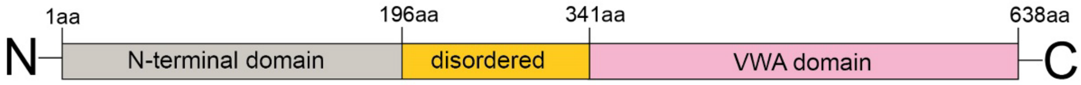

Figure S5: Prediction of disordered regions and overall architecture for full length NorD from P. denitrificans. *a) Disordered regions predicted for NorD. b) Overview of the architecture of the NorD protein including the N-terminal domain (grey), disordered linker (yellow) and the C-terminal VWA-domain (pink) as predicted by AlphaFold (see Fig. 7a in main text).*

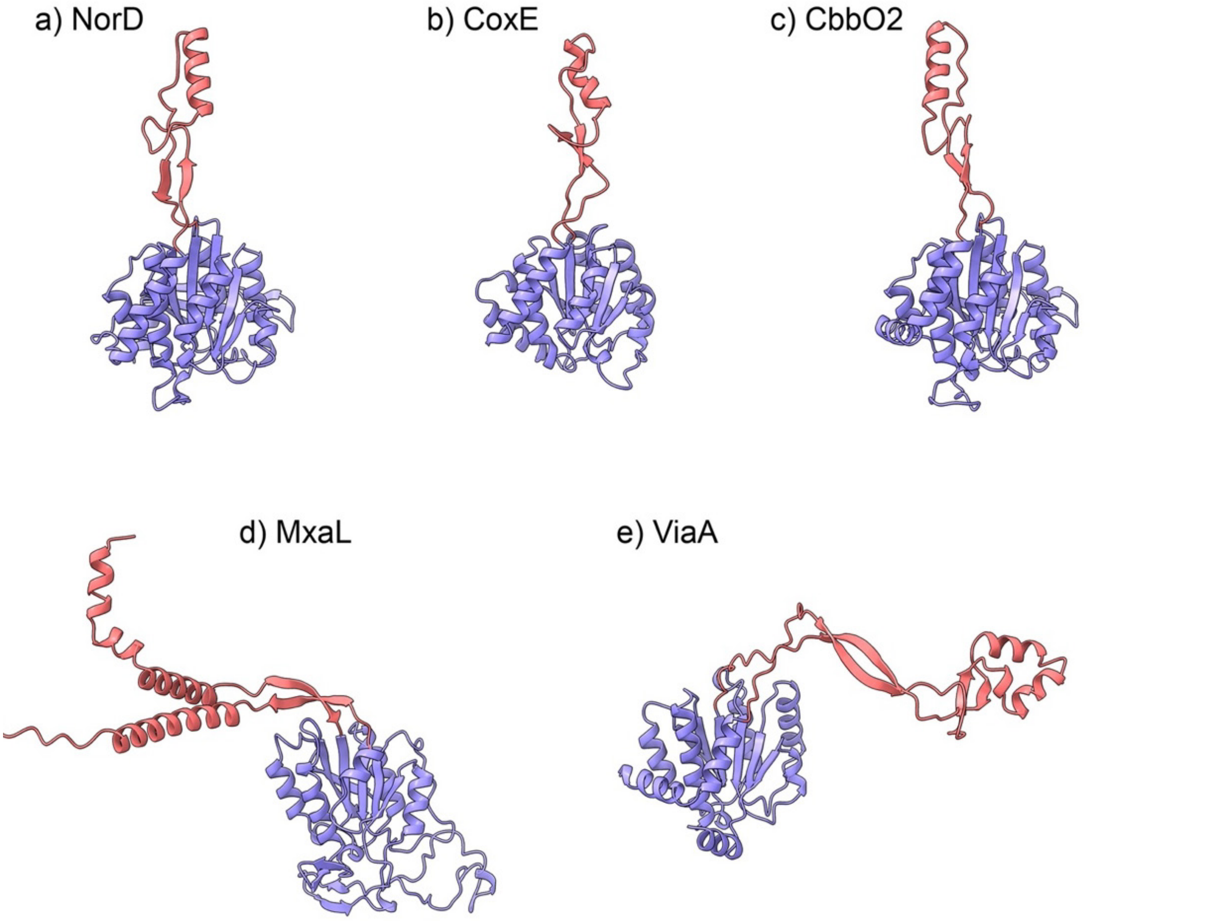

protein (VWA domain only)	*Uniprot ID*	*residues VWA*	*sequence identity (%)*	*sequence similarity (%)*
NorD	*Q51665*	***337-638***	***100***	***100***
CoxE	*Q9KX26*	***163-399***	***18***	***28***
CbbO2	*B7J5E5*	***463-759***	***31***	***51***
MxaL	*C5AQA0*	***1-336***	***12***	***21***
ViaA	*P0ADN0*	***195-483***	***18***	***30***

Figure S6**: **Finger-like protrusions predicted for several MoxR-associated VWA domains and pairwise alignments. *The predicted VWA domains are all models produced by AlphaFold. VWA domains in blue and finger-like protrusions in red. a) NorD from P. denitrificans (Uniprot: Q51665), b) CoxE from Afipia carboxidovorans (Uniprot: Q9KX26), c) CbbO2 from Acidithiobacillus ferrooxidans (B7J5E5), d) MxaL from Methylorubrum extorquens (Uniprot: C5AQA0), e) ViaA from E. coli (Uniprot: P0ADN0). The table shows the results of pairwise alignments of the NorD VWA domain to the other VWA domains depicted in this figure. The models shown correspond to the sequence fragments used for the alignments.*

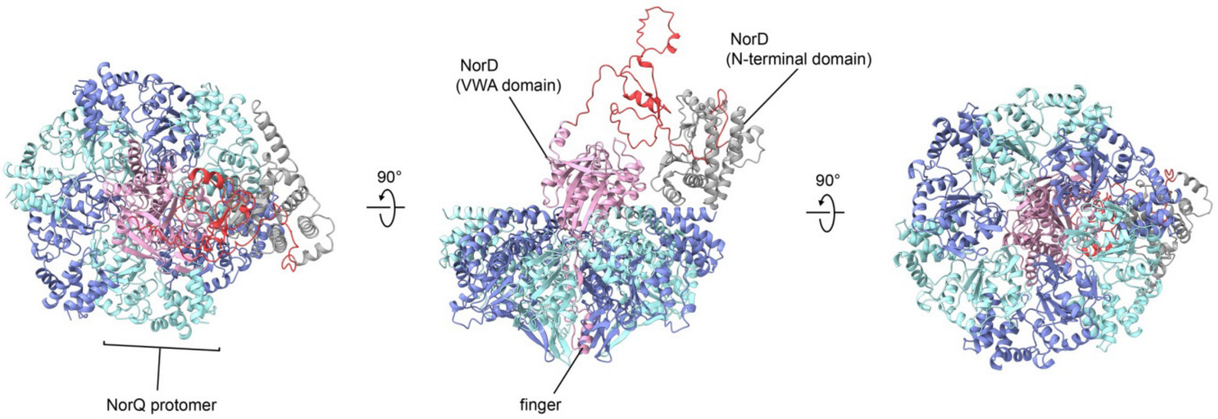

Figure S7: AlphaFold multimer model of the full length NorD in complex with the NorQ hexamer. *One NorD chain modelled together with 6 NorQ chains. The NorQ protomers are colored in cyan and blue. The NorD is shown with the N-terminal domain in grey, the disordered region in red and the VWA domain in pink. The 'finger' structure of the VWA domain located in the central pore of the NorQ hexamer is marked.*

Table S2: AlphaFold multimer prediction datasets. *The predictions are listed together with their corresponding pTM scores. AF-m=AlphaFold multimer.*

**Model**	**Average pTM**	**used for figure**	**Description**	**# models obtained**	**AF-m release**
NorD_Nterm-NorQ	0.39	not shown	N-terminal domain of NorD with the NorQ hexamer	5	v2.1.1
NorD_VWA-NorQ	0.49	Figure 7d	C-terminal (VWA domain) of NorD and the NorQ hexamer	5	v2.1.1
NorD-NorQx1-split	0.69	Figure 7c	N-terminal and C-terminal domains of NorD, without disordered domain, with NorQ monomer	5	v2.1.1
NorD-NorQ_v2	0.41	Figure S9	NorD monomer plus NorQ hexamer	5	v2.2.0
NorD-NorQx1	0.70	Figure 7b	NorD and NorQ monomer each	1	v2.2.0

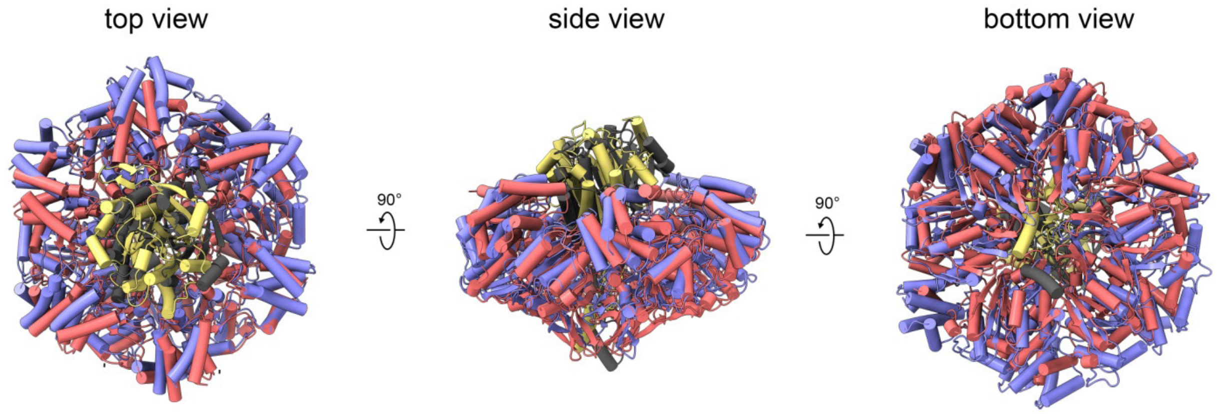

Figure S8: Superposition of the NorQ-VWA AlphaFold prediction and the fitted cryoEM-model. *The figure shows the superposition of the prediction of NorQ*$_6$*VWA*$_1$ *(Fig. 7d) and the fitted model based on our cryo-EM map of the NorQD complex (Fig. 8a). The NorQ*$_6$*VWA*$_1$ *AlphaFold model is shown in red (NorQ) and yellow (VWA) and our fitted model is in blue (NorQ) and black (VWA).*

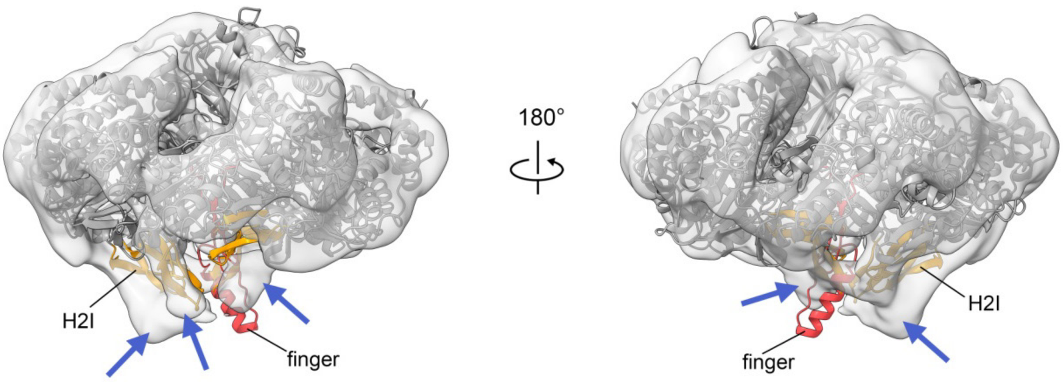

Figure S9*: *Extra densities in the NorQ^{WB}D cryo-EM map. *Shown is the modeled complex of the NorQ hexamer together with NorD VWA domain as fitted into the cryo-EM map (see main text Fig. 8). Extra densities that were not well fitted on the convex side of the NorQ ring are marked with blue arrows. The extra densities are in close proximity to the H2Is of the NorQ protomers (yellow) and the 'finger' structure (red) protruding from the NorD VWA domain.*

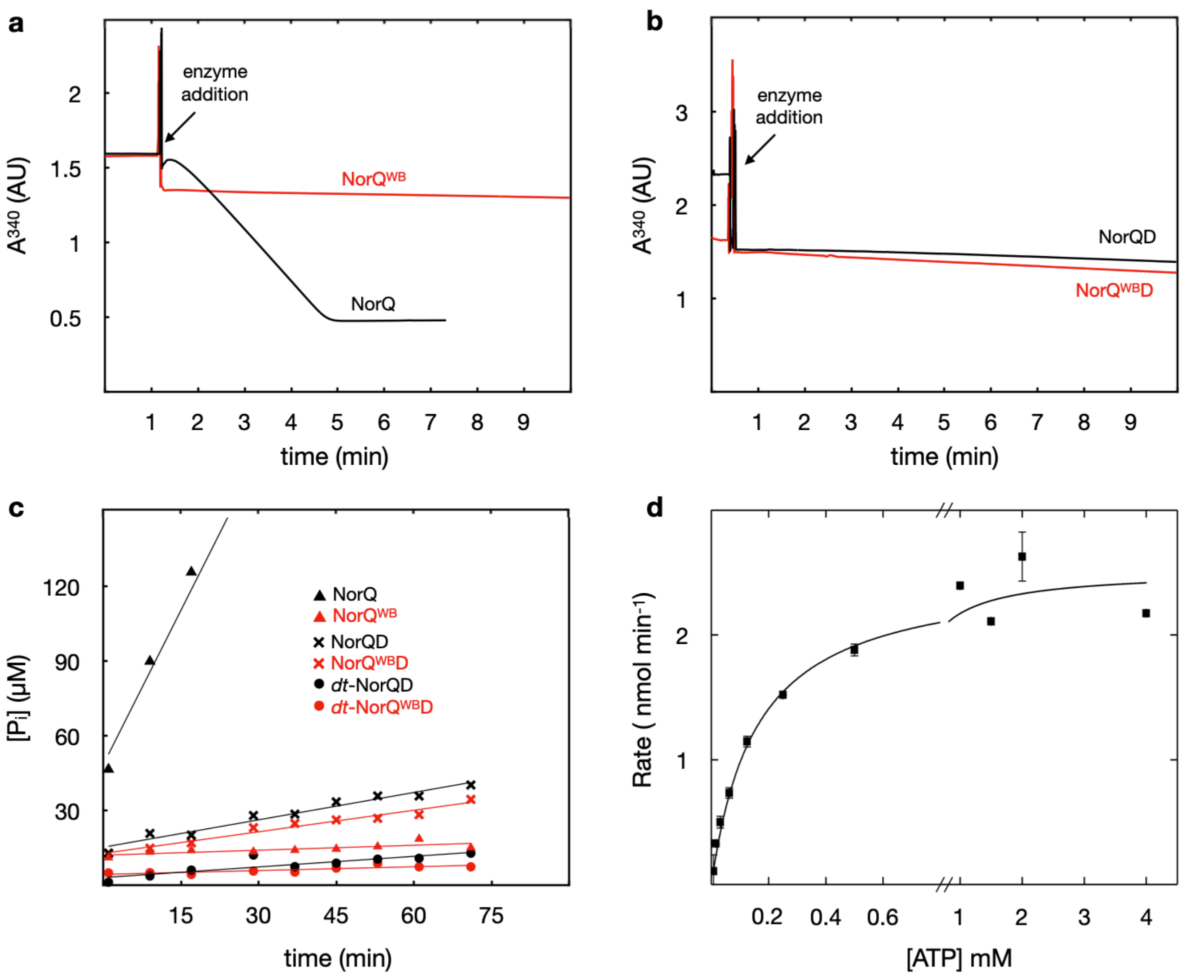

Figure S10: ATPase activity measurements. *a) NorQ (black) and NorQ^{WB} (red) in the NADH-coupled assay. b) NorQD (black) and NorQ^{WB}D (red) in the NADH-coupled assay. The reaction mixture contained 50 mM TRIS/HCl, pH 8.0, 150 mM NaCl, 15 mM $MgCl_2$, 2.5 mM ATP, 1 mM phosphoenolpyruvate, 0.3 mM NADH, 12 U/mL pyruvate kinase, 12 U/mL lactate dehydrogenase. c) Linear regressions for data from the malachite green end-point assay for NorQ (black triangles), NorQ^{WB} (red triangles), NorQD (black crosses), NorQ^{WB}D (red crosses), double tagged NorQD (black circles) and double tagged NorQ^{WB}D (red circles). The initial reaction mixture contained 50 mM TRIS/HCl, pH 8, 150 mM NaCl, 10% (v/v) glycerol, 20 mM $MgCl_2$, 2 mM ATP. Data shown in A-C are example traces. d) Michaelis-Menten kinetics for NorQ (0.15 mg/ml). The data (shown as the average ± SD, n=2) was fitted to a K_m=0.16±0.02 mM, V_{max}=2.5±0.1 nmol min^{-1} and a Hill coefficient = 1. The corresponding k_{cat} is 20±1 ATP min^{-1} hexameric complex^{-1}. For data, see additional file 4.*

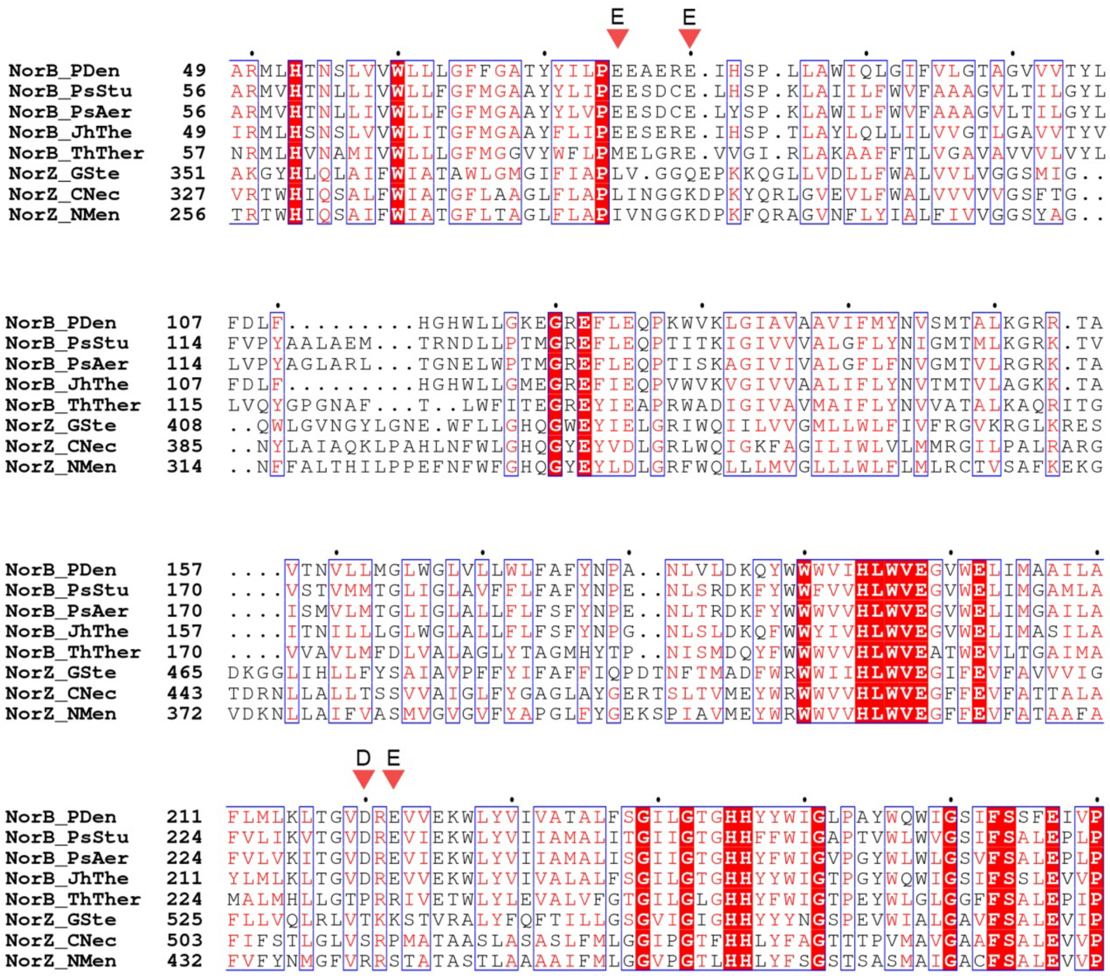

Figure S11: Sequence alignment of the NorB subunit of cNOR with NorZ (qNOR) from various organisms. *We included cNOR from P. denitrificans (PDen; Uniprot: Q51663), Ps. stutzeri (PsStu; Uniprot: P98008), Ps. aeruginosa (PsAer; Uniprot: A0A072ZET2), Jhaorihella thermophila (JhThe; NCBI: WP_104006941.1) and Thermus thermophilus (ThTher; Uniprot: D5GU63). Sequences of qNOR are from G. stearothermophilus (GSte; B3Y963), Cupriavidus necator (CNec; O30375) and Neisseria meningitides (NMen; Q9JPL2). The acidic residues that were found conserved on the cytoplasmic surface of cNOR (excluding the T. thermophilus cNOR) but not in qNOR and that were mutated in this study are marked by red triangles.*

Table S3: Re-activation experiments with cNOR using crude cell extracts. *Data presented as the average value with standard error (n=2). For data, see Additional file 4.*

Genes present (soluble fraction)	***Genes present (membrane fraction)***	***Activity of purified cNOR (%)***	***Non-heme Fe content (%)***
norCBQDEF	*norCBQDEF*	*100 ± 20*	*100 ± 10*
norCBQDEF	*norCB(ΔQDEF)*	*26 ± 1*	*21 ± 1*
norCB(ΔQDEF)	*norCB(ΔQDEF)*	*9 ± 3*	*10 ± 1*

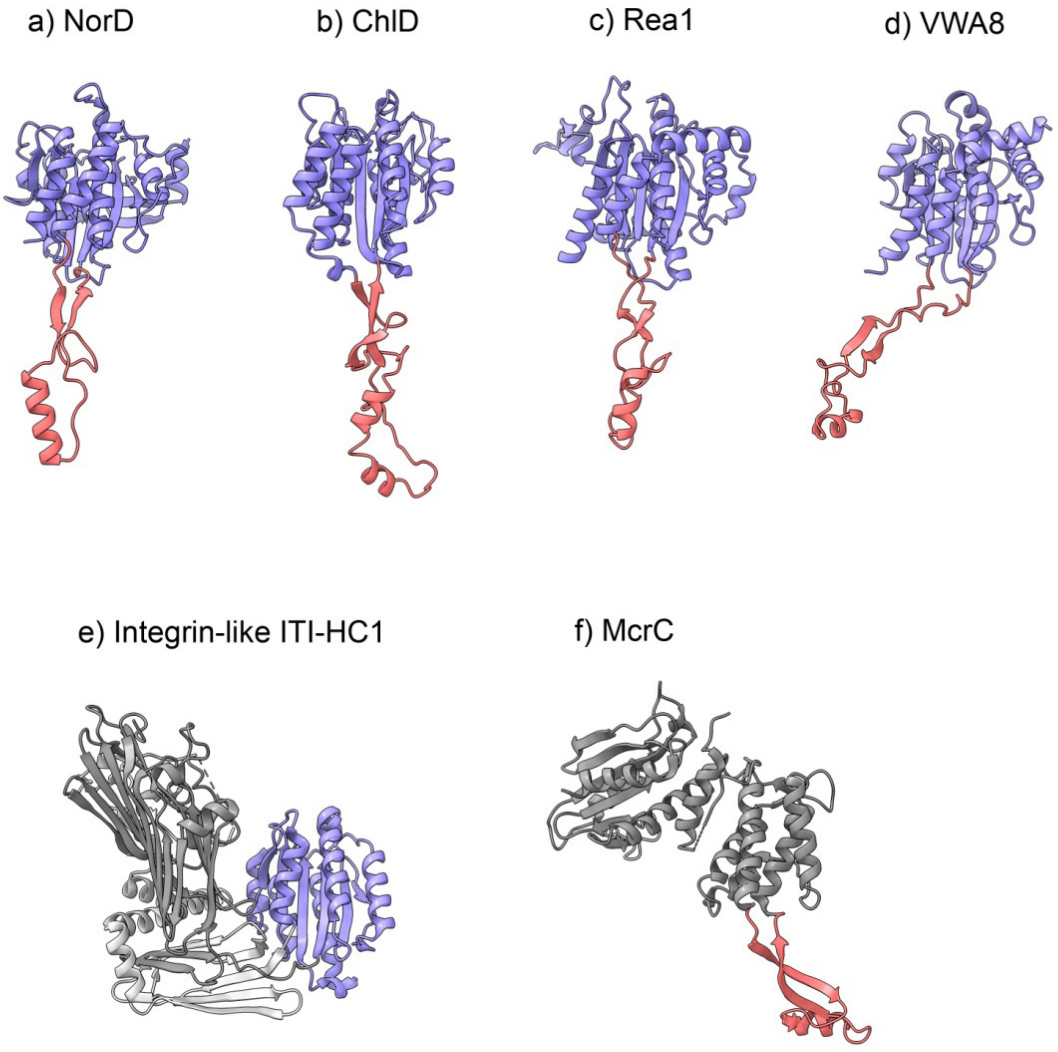

***Figure S12**: **Predicted VWA domain of NorD in comparison to several eukaryotic VWA domains.** a-e) VWA domains, f) McrC. The predicted VWA domains (a-d) are all models produced by AlphaFold. VWA domains in blue and finger-like protrusions in red. Remaining structural elements are colored in grey. a) NorD from P. denitrificans (Uniprot: Q51665), b) ChlD from Arabidopsis thaliana (Uniprot: Q9SJE1), c) Rea1 from Saccharomyces cerevisiae (Uniprot: Q12019), d) human VWA8 (Uniprot: A3KMH1), e) crystal structure of a human ITI-HC1 monomer (PDB: 6FPY). f) cryo-EM structure of McrC from E. coli (Uniprot: P15006, PDB: 6HZ4).*

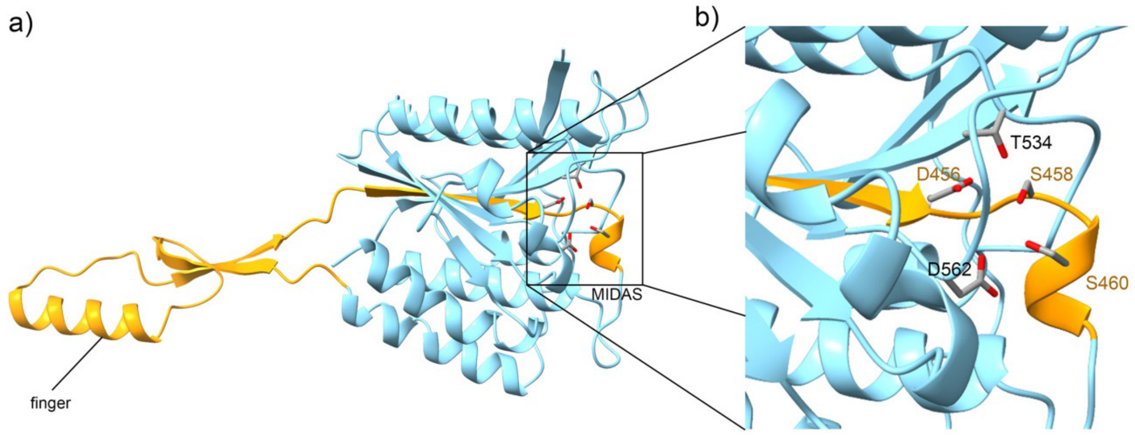

Figure S13: Structural connection between the 'finger' and the MIDAS site in the NorD VWA domain. *The figure shows the AlphaFold prediction model of NorD. a) The 'finger' and MIDAS are positioned at opposite sides of the VWA domain, but a single β-strand (orange) connects the 'finger' to the MIDAS residues D456, S458 and S460 located in the VWA core (cyan). b) Close-up of the MIDAS, with the residues indirectly connected to the 'finger' via a single β-strand labeled in orange.*

Table S4: Primers used in this study.

function	forward primer	reverse primer
N-terminal His tag NorQ in pNOREX	CGGAGTAAGCCATGCATCATCATCATCATCATAACGCGCATGTGAAAACCCAAGGG	CACATGCGCGTTATGATGATGATGATGATGCATGGCTTACTCCGCAGCCTCC
N-terminal His tag NorD in pNOREX	GGGAAGCGCCATGCATCATCATCATCATCATGGTCTGGACCTGGAACCC	GGTCCAGACCATGATGATGATGATGATGCATGGCGCTTCCCCTCAGCC
N-terminal His tag NorQ in pET21	GAGATGCGCGTTGCTAGCATGATGATGATGATGATGCATATGTATATCTCCTTCTTAAAGTTAAAC	CACATGCGCGTTGCTAGCATGATGATGATGATGATGCATATGTATATCTCCTTCTTAAAGTTAAAC
N-terminal His tag NorD in pET21	CGCCATGCATCATCATCATCATCATTCTAGAGGTCTGGACCTGGAACCCTGGGAGC	CCAGACCTCTAGAATGATGATGATGATGATGCATGGCGCTTCCCCTCAGCCGAA
Cloning NorQ into pETDuet-1	TGAGATCTCAACGCGCATGTGAAAACCC	TGATCTCGAGGCCGAAGATCGCGGCG
Cloning NorD into pETDuet-1	ATCGAATTCGGGTCTGGACCTGGAACCC	GATAAGCTTTCACGCCACCAGTTGCCGATAG
Exchange of S-tag for Strep tag II in pETDuet-1	ACTGCTCGAGAGCGCTTGGAGCCACCCGCAGTTCGAAAAATGACCATGGTTAATTAATGA	TCATTAATTAACCATGGTCATTTTTCGAACTGCGGGTGGCTCCAAGCGCTCTCGAGCAGT
Amplifying NorQ and adding restriction sites	AGAGCTAGCAACGCGCATGTGAAAACCC	TGTCTGAATTCCTTCCCCTCAGCCGAAGATC
Amplifying NorD and adding RBS and restriction sites	AGCGAATTCAAGAAGGAGATATACATATGCATCATCATCATCATCATTCTAGAGG	TCTAAGCTTTCACGCCACCAGTTGCC
Walker B mutation in NorQ (E109Q)	CTCGACCAGGTGGTCGAGGCGCGCAAGGACG	GACCACCTGGTCGAGATAGCAGATCGCGCCCTCG
Walker A mutation in NorQ (K48A)	GCGGCGCGACCCGCTTCGTCGCCCATATGG	GCGGGTCGCGCCGCAGCCGGTCGGGCCTTTCAGC
T534V mutation in NorD	GCGCTTCTATGTGCGGCTTGGCGCGG	CCAAGCCGCACATAGAAGCGCGGCCTCAGC
D562N mutation in NorD	GGTCATCACCAACGGCAAGCCCAACGACCTGG	GCTTGCCGTTGGTGATGACCAGAAGCAGCCG
E75A mutation in NorB	GCCCGCAGAGGCCGAGCGCGAGATCCATTCGCC	GCCTCTGCGGGCAGGATGTAATAGGTCGCGCCG
E78A mutation in NorB	GGCCGCGCGAGTCCATTCGCCGCTGC	GCGCGCGGCCTCTTCGGGCAGGATGTAATAGGTC
Double mutation E75A and E78A in NorB	CCCGCAGAGGCAGCGCGCGAGATCCATTCGCCGCTG	GCGCGCTGCCTCTGCGGGCAGGATGTAATAGGTCGC
D220A mutation in NorB	CGTGGCCCGCGAGGTGGTCGAGAAATGGCTTTACGT	CGCGGGCCACGCCGGTCAGCTTGAGCATCA
