# Supplementary material for: Insights into the structure-function relationship of the NorQ/NorD chaperones from Paracoccus denitrificans reveal shared principles of interacting MoxR AAA+/VWA domain proteins
Source: BMC Biol. 2023 Feb 28;21:47. doi: 10.1186/s12915-023-01546-w (PMC9976466; doi:10.1186/s12915-023-01546-w)
Supplement: Supplementary file 2 — Additional file 2. Original BN-PAGE gels, related to Figs. 2 and 3. [file 12915_2023_1546_MOESM2_ESM.pdf]

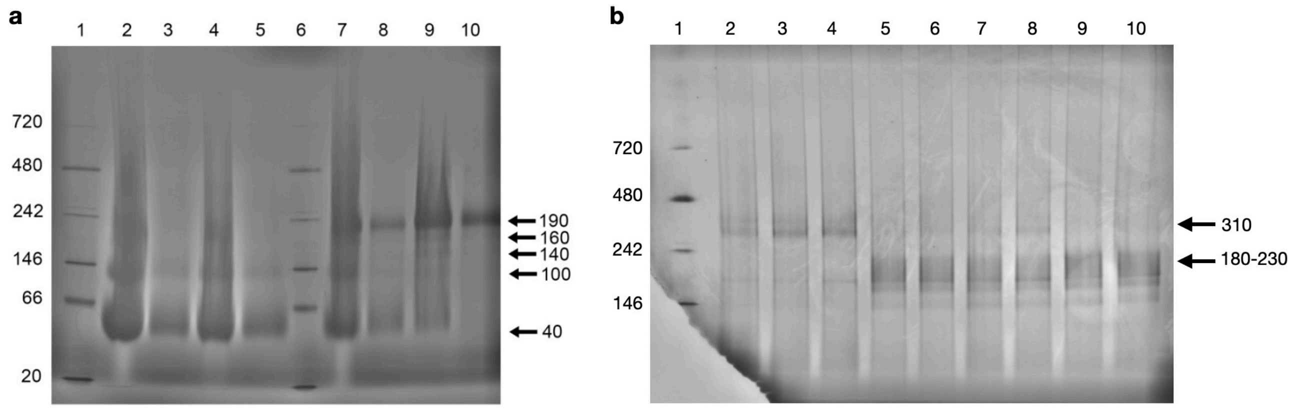

Blue Native (BN-) PAGE original gels. *a) Blue Native (BN-) PAGE of wild type NorQ and NorQ^{WB} with and without addition of ATP and $MgCl_2$. Original gel to Figure 2b in main text. Lane 1: Native Marker, 5 µL. Lane 2: NorQ, 29 µg. Lane 3: NorQ, 2.9 µg. Lane 4: NorQ, 14 µg, with 2 mM ATP and 20 mM $MgCl_2$, Lane 5: NorQ, 2.9 µg, with 2 mM ATP and 20 mM $MgCl_2$. Lane 6: Native Marker, 5 µL. Lane 7: NorQ^{WB}, 26 µg. Lane 8: NorQ^{WB}, 3.6 µg, Lane 9: NorQ^{WB}, 18 µg with 2 mM ATP and 20 mM $MgCl_2$. Lane 10: NorQ^{WB}, 3.6 µg with 2 mM ATP and 20 mM $MgCl_2$. Gel run for 1h at 150 V and 45 min at 250 V. b) Blue Native (BN-) PAGE of NorQ^{WB}D with different additions of ATP and $MgCl_2$. Original gel to Figure 3b in main text. Lane 1: Native Marker, 5 µL. Lane 2-4: NorQ^{WB}D, 10 µg, with 20 mM $MgCl_2$. Lane 5-6: NorQ^{WB}D, 10 µg, with 0.5 mM ATP and 20 mM $MgCl_2$. Lane 7-8: NorQ^{WB}D, 10 µg, with 1 mM ATP and 20 mM $MgCl_2$. Lane 9-10: NorQ^{WB}D, 10 µg, with 2 mM ATP and 20 mM $MgCl_2$. Run at 4° C for 1 h at 150 V, followed by 30 min at 250 V.*
