# Supplementary material for: Insights into the structure-function relationship of the NorQ/NorD chaperones from Paracoccus denitrificans reveal shared principles of interacting MoxR AAA+/VWA domain proteins
Source: BMC Biol. 2023 Feb 28;21:47. doi: 10.1186/s12915-023-01546-w (PMC9976466; doi:10.1186/s12915-023-01546-w)
Supplement: Supplementary file 3 — Additional file 3. Original SDS-PAGE gels. [file 12915_2023_1546_MOESM3_ESM.pdf]

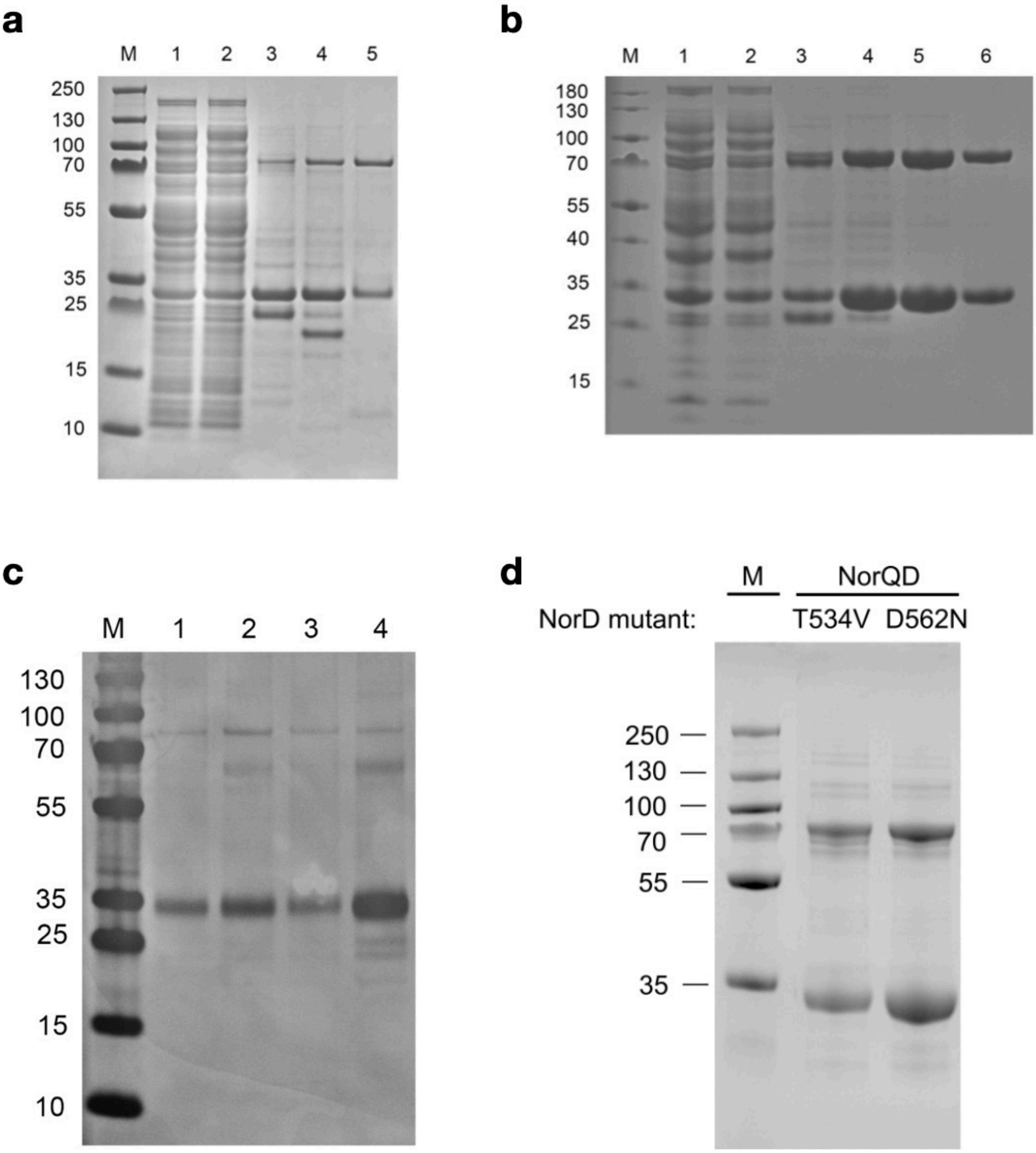

Original SDS-PAGE gels. *a) SDS-PAGE of NorQD purification. Original gel to part of Figure 3a in main text. Lane M: PageRuler Plus Prestained, 5 µL. Lane 1: Cytosolic suspension. Lane 2: Flow-through from Ni-NTA. Lanes 3-5; fractions from imidazole stepwise elution: 3: 150 mM imidazole, first peak. Lane 4: 150 mM imidazole, second peak. Lane 5: 200 mM imidazole. b) SDS-PAGE of NorQ^{WB}D purification. Original gel to part of Figure 3a in main text. Lane M: PageRuler Prestained. Lane 1: Cytosolic suspension. Lane 2: Flow-through from Ni-NTA. Lanes 3-6: Fractions from the imidazole gradient: Lane 3: 100-160 mM imidazole. Lane 4: 160-210 mM imidazole. Lane 5: 210-260 mM imidazole. Lane 6: 260-360 mM imidazole. c) SDS-PAGE on BN-PAGE bands (Additional File 2b). Original gel to Figure 3c in main text. Lane M: PageRuler Plus Prestained. Lane 1: Upper 310 kDa band from lanes 2-4 in Additional file 2b. Lane 2: Lower 310 kDa band from lanes 2-4 in Additional file 2b. Lane 3: 180-230 kDa band from lanes 2-4 Additional file 2b. Lane 4: 180-230 kDa band from lanes 5-10 Additional file 2b. d) SDS PAGE of co-purified NorQD after mutagenesis of the conserved MIDAS motif. Lane 1: PageRuler Plus Prestained. Lane 2: NorQD^{T534V}, 1 µg. Lane 3: NorQD^{D562N}, 1 µg. All gels run in room temperature at 100 V.*
