# Supplementary material for: Insights into the structure-function relationship of the NorQ/NorD chaperones from Paracoccus denitrificans reveal shared principles of interacting MoxR AAA+/VWA domain proteins
Source: BMC Biol. 2023 Feb 28;21:47. doi: 10.1186/s12915-023-01546-w (PMC9976466; doi:10.1186/s12915-023-01546-w)
Supplement: Supplementary file 4 — Additional file 4. Data related to Tables 1 and 2, Additional file 1: Fig. S10 and Table S3. [file 12915_2023_1546_MOESM4_ESM.pdf]

NADH-coupled assay

Sample	NorQ	$NorQ^{WB}$	NorQD	$NorQ^{WB}D$	dt-NorQD	dt-$NorQ^{WB}D$		
Rate 1 (nmol min^{-1} mg^{-1})	154.905268817204	1.24354838709677	31.329806451613	12.823494623656	1.32104301075269	0.228696129032258		
Rate 2 (nmol min^{-1} mg^{-1})	139.241827956989	1.48118064516129	28.9177634408603	13.281623655914	4.51735268817204	0.762118924731182		
Rate 3 (nmol min^{-1} mg^{-1})	127.930860215054	1.82465913978495	29.0990107526882	12.3952150537635		1.74448279569893		
Mean rate (nmol min^{-1} mg^{-1})	140.692652329749	1.51646272401434	29.7821935483872	12.8334444444445	2.91919784946237	0.91176594982079		
SE (nmol min^{-1} mg^{-1})	7.82055727723163	0.168677251517605	0.775573319268726	0.255932479176242	1.59815483870967	0.443921156212731		

Malachite green assay

Sample	NorQ	$NorQ^{WB}$	NorQD	$NorQ^{WB}D$	dt-NorQD	dt-$NorQ^{WB}D$		
Rate 1 (nmol min^{-1} mg^{-1})	72.72	1.82	7.34	7.33	2.85	1.024		
Rate 2 (nmol min^{-1} mg^{-1})	75.733	1.334	9.94	5.78	4.656	1.64		
Mean rate (nmol min^{-1} mg^{-1})	74.2265	1.577	8.64	6.555	3.753	1.332		
SE (nmol min^{-1} mg^{-1})	1.5065	0.243	1.3	0.775	0.903	0.308		

cNOR activity normalized to % of WT

Sample	E109 (NorQ)	T534V (NorD)	D562N (NorD)	E75/E78A (NorB)	D220A (NorB)	E222A (NorB)	N-terminal His-tag (NorD)	N-terminal His-tag (NorQ)
Rate 1 (%)	6.2	4.5	3.4	32	7.1	5.1	63	61
Rate 2 (%)	0.4	0.2	4.7	51	1.8	2	65	66
Rate 3 (%)				36			80	110
Rate 4 (%)				24				
Rate 5 (%)				72				
Rate 6 (%)				34				
Mean (%)	3.3	2.35	4.05	41.5	4.45	3.55	69.3333333333333	79
SE (%)	2.9	2.15	0.65	7.07931258433096	2.65	1.55	5.36449231314369	15.5670592384475

Non-heme Fe content normalized to % of WT

Sample	D220A	E222A						
Amount of iron 1 (%)	33.8673813473344	32.428326268108						
Amount of iron 2 (%)	28.5239461762003	33.2475426573204						
Amount of iron 3 (%)	22.0293714996756	7.12514351616156						
Mean (%)	28.1402330077368	24.2670041471967						
SE (%)	3.42272046453577	8.57419225143029						

Re-activation cNOR activity

Sample	*norCBQD+norCBQD*	*norCBQD+norCB*	*norCB+norCB*					
Rate 1 (e s^{-1})	6.6	1.6	0.4					
Rate 2 (e s^{-1})	5.2	1.5	0.65					
Mean (e s^{-1})	5.9	1.55	0.525					
SE (e s^{-1})	0.7	0.05	0.125					

Re-activation non-heme Fe content

Sample	*norCBQD (so)+ norCBQD (m)*	*norCBQD+norCB*	*norCB+norCB*					
ΔA593 1	0.078	0.015	0.008					
ΔA593 2	0.065	0.014	0.007					
Mean	0.0715	0.0145	0.0075					
SE	0.0065	0.0005	0.0005					
